# An Evolutionary View of *Trypanosoma Cruzi* Telomeres

**DOI:** 10.3389/fcimb.2019.00439

**Published:** 2020-01-10

**Authors:** Jose Luis Ramirez

**Affiliations:** Fundación Instituto de Estudios Avanzados and United Nations University UNU-BIOLAC, Caracas, Venezuela

**Keywords:** *Trypanosoma cruzi* (*T. cruzi*), telomeres, evolutioanry dyanmics, genome, sialidases

## Abstract

Like in most eukaryotes, the linear chromosomes of *Trypanosoma cruzi* end in a nucleoprotein structure called the telomere, which is preceded by regions of variable length called subtelomeres. Together telomeres and subtelomeres are dynamic sites where DNA sequence rearrangements can occur without compromising essential interstitial genes or chromosomal synteny. Good examples of subtelomeres involvement are the expansion of human olfactory receptors genes, variant surface antigens in *Trypanosoma brucei*, and *Saccharomyces cerevisiae* mating types. *T. cruzi* telomeres are made of long stretches of the hexameric repeat 5′-TTAGGG-OH-3′, and its subtelomeres are enriched in genes and pseudogenes from the large gene families RHS, TS and DGF1, DEAD/H-RNA helicase and N-acetyltransferase, intermingled with sequences of retrotransposons elements. In particular, members of the Trans-sialidase type II family appear to have played a role in shaping the current *T. cruzi* telomere structure. Although the structure and function of *T. cruzi* telomeric and subtelomeric regions have been documented, recent experiments are providing new insights into *T. cruzi*'s telomere-subtelomere dynamics. In this review, I discuss the co-evolution of telomere, subtelomeres and the TS gene family, and the role that these regions may have played in shaping *T. cruzi*'s genome.

## Introduction

*Trypanosome cruzi* causes Chagas disease a debilitating and often lethal malaise affecting millions of people in Latin American countries. *T. cruzi* populations are very variable and this variability is due in part to a clonal population structure, genome plasticity and abundance of repeated sequences. Nearly 50% of the total genome is made of repeated sequences, some of which code for protein families such as the Transialidases (TS), mucins (MUC), mucins associated proteins (MASP), Disperse gene family 1 (DGF-1), Retrotransposon Hot Spot proteins, and retrotransposons elements (El-Sayed et al., 2005).

The Trans-sialidase family is divided into eight groups (Freitas et al., 2011), and the family name derives from enzymes included in the first group that can transfer host sialic acid onto the parasite's surface MUC. The rest of the TS groups have no enzymatic activity but code for surface glycoproteins involved in infectivity, adhesion, and evasion from the host immune response. MUC are not only receptors for sialic acid (Di Noia et al., 1995) but together with MASP proteins (Bartholomeu et al., 2009) provide a developmentally regulated shield that protects the parasite in hostile environments. DGF-1 are integrin-like proteins (Gonzalez et al., 2009; Kawashita et al., 2009; Lander et al., 2010) that are developmentally regulated, but whose role has not been deciphered. The Retrotransposon Hot Spot (RHS) family codes in *T. brucei* for nuclear and perinuclear proteins and their sequences are targets for the insertion of RIME/ingi non-LTR retrotransposons (Bringaud et al., 2002). In *T. cruzi* RHS genes are frequently interrupted by the non-LTR retrotransposon LTc1 (Bringaud et al., 2006).

Early experiments in Manning's lab (Peterson et al., 1989; Ruef et al., 1994) revealed that two members of the Transialidase family type II (TSII) located in the vicinity of the telomere were highly expressed, whereas another member, in a more internal chromosomal location, showed a lower expression level (Peterson et al., 1989). After this original report, Freitas-Junior et al. (1999) and Chiurillo et al. (1999) confirmed the presence of TSII gene members in the subtelomeres of several *T. cruzi* strains. The cloning of *T. cruzi*'*s* telomeric and subtelomeric regions using a vector-adaptor complementing the last nine nucleotides of the telomere (Chiurillo et al., 1999, 2002; Kim et al., 2005), allowed us to define the sequence of the telomeric repeat, and have a detailed view of a large segment of the subtelomere.

Subtelomeres are often described as regions of genomic instability (Baird, 2018), and this observation is supported by the abundance of retrotransposon fragments and pseudogenes in *T. cruzi* telomeric clones, but they also represent a buffer region to minimize chromosome damage when telomere attrition occurs. However, despite this unstable environment, complete gene copies of DGF-1, TS, and RHDEAD/H-RNA helicase and N-acetyltransferase in *T. cruzi* subtelomeres are expressed, indicating that a positive selection is preserving the integrity of these genes at these locations (Moraes Barros et al., 2012). At the end of subtelomeres, there is a 189 base pairs sequence with homology for the 3′ and 5′UTRs sequences of a gp85 gene (TSII) that we dubbed the 189 bp junction (Kim et al., 2005). A recapitulation of the possible events associated with the creation of this junction was proposed by Kim et al. (2005), Chiurillo et al. (2017) in which tandem repeats of TSII genes suffered breakages, excisions and rejoining to generate the junction. After this junction, the chromosomes are capped by runs of hexameric repeats of variable length ending in a single strand terminus 5′-TTAGGG-OH-3′.

## The Evolution of *T. cruzi* Telomeres and the Role of Members of the TSII Gene Family in Shaping its Genome

The intimate association between telomeres and TSII (gp85) sequences raises many questions about the creation of *T. cruzi* telomeres, such as how and when TS II family members were incorporated into the 189 bp junction? Were they part of transposon elements that eventually evolved into a primitive telomere? Did they come along with the telomeric repeats?

When we examine the structure of eukaryotic telomeres we observe a tremendous diversity in telomeric repeats and sequences associated with telomeres, the extreme case of Diptera where transposons assumed the role of telomeres. Telomeres arose during eukaryogenesis as a need to protect the ends of linear chromosomes from exonucleases degradation, prevent chromosome rearrangements, and facilitate the replication of the DNA lagging strand. This process may have started with the endosymbiosis of an ancestral phagotrophic host cell (Cavalier-Smith, 2002) and an α-proteobacterium carrying Type II introns. Type II introns are Eubacterial mobile retroelements with ribozyme and reverse transcriptase activities. Eventually, Type II introns were transferred into the host cell genome and gave rise to the spliceosome, the non-LTR transposons and telomerase (Garavis et al., 2013; Podlevsky and Chen, 2016). According to this hypothesis, these non-LTR transposons were inserted at several genome locations, including the chromosome ends, originating the prototelomeres.

On the other hand, although still debatable, based on the sequence homogeneity of the transialidases within the animal kingdom, and their sequence homology with their very variable bacterial counterparts (Roggentin et al., 1993) proposed that sialidases originated in ancestors of the Echinodermata and Deuterostomate animals, and later these genes were horizontally transferred to bacteria via viruses. However, Schwerdtfeger and Melzig (2010) argued that the irregular appearance of sialidases in invertebrates does not support a common evolution of this gene family. In agreement with this view, in the *Trypanosomatidae* family, a taxon that includes pathogenic flagellates, we also observe this kind of patchy inheritance, since of all genera within this family the TS genes are only present in the genus *Trypanosoma*.

It is assumed that the first trypanosome to acquire sialidase genes was *T. brucei* or a common ancestor of both *T. brucei and T. cruzi*. The separation *Trypanosoma brucei* from *Trypanosoma cruzi* occurred approximately 100 millions of years during the Gondwanaland breakage (Briones et al., 1995; Stevens et al., 1999), and since then, the TS family suffered an expansion exclusively in the *T. cruzi* clade (Chiurillo et al., 2016a). During this long separation, the two species developed different survival strategies within their vectors and hosts. *T. brucei* uses the telomeric-subtelomeric compartment as a specialized expression site for variable antigenic determinants. On the contrary, *T. cruzi* acts like a stealth invader jamming the vertebrate host immune system through a simultaneous expression of multiple surface antigens (Millar et al., 1999), and among these, members of the TS family play an important role.

**Figure 1:**
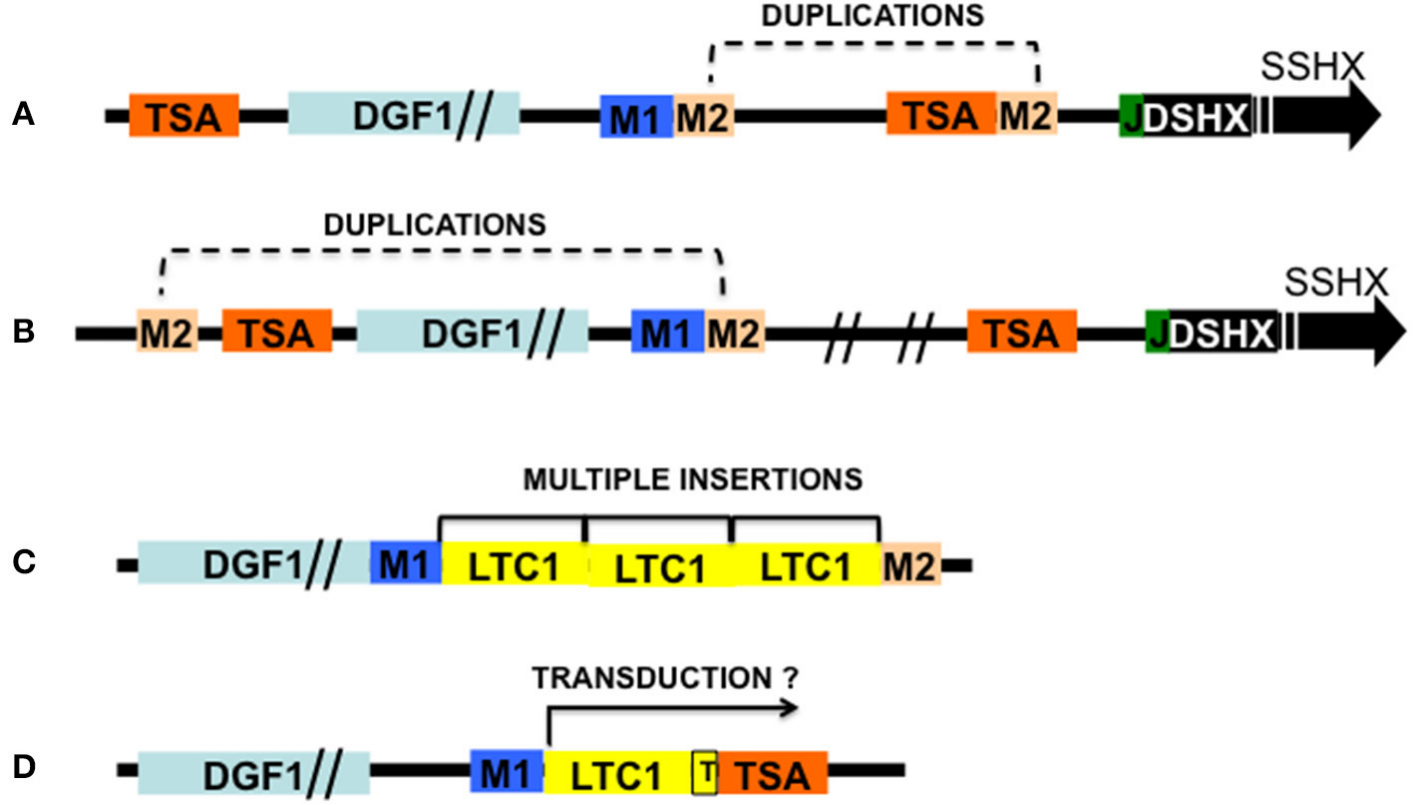
Schematic representation of *T. cruzi* telomeric recombinants and two recombinants containing retrotransposons associated sequences. Modules M1 and M2 are LTc1 retrotransposon flaking sequences: M1, RS13TC+RS1Tc (upstream); M2, Seq3Tc + SIRE associated sequences SZ (downstream); TSA, transialidase genes (or pseudogenes); L1Tc, LTc1 non-LTR retrotransposon (genes and pseudogenes); DGF1, Disperse gene family 1 genes (or pseudogenes); J, 189 bp junction; DSHX, double strand hexameric sequences; SSHX, single strand hexameric sequences. **(A)** Telomeric BAC6 recombinant containing *T. cruzi* subtelomere and telomere showing duplication of M2. **(B)** Telomeric cosmid C6 showing M2 duplications. **(C)** Recombinant pBAC62 showing multiple LTc1 retrotransposons inserted between M1 and M2. **(D)** Recombinant pBAC52 showing a TSA gene located downstream of 213 bp of a truncated LTc1 (T) flanked upstream by the insertion sequence GTATCTTTG and a poly A track. Probably the TSA gene was transducted by the retrotransposon. Sizes are approximated; the Flanking sequences are part of the Retrotransposon Hot Spot proteins. Adapted from Kim et al. (2005), Olivares et al. (2013).

How the expansion of TSII genes occurred in *T. cruzi* is unknown, but likely, several events of gene mutation, duplication, recombination, transposition, and genetic drift generated the current picture of the TS family. Vestiges of these events are the salad of sequences derived from retrotransposon elements and Retrotransposons Hot Spot (RHS) genes scattered through the genome, and particularly in *T. cruzi* subtelomeres (Figure 1). The abundance of these elements at the subtelomere suggests the active participation of these regions in shaping the parasite genome (Kim et al., 2005). Regarding the telomeric repeat 5′-TTAGGG-OH 3′, it is found in Plantae, Chromoalveolata, Excavata (where Trypanosomatids are included) and Rhizaria supergroups, a reason why some authors (Dressen et al., 2007; Fulneckova et al., 2013) have suggested that it was the repeat of the ancestral Eukaryotic telomeres. Thus, a safe assumption is that the TS sequences in the subtelomeric transition (189 bp junction) appeared after the hexameric repeats were already fixed, and perhaps the fixation of the 189 bp junction has to do with its adaptation to the telomeric function, thus this sequence appears to be an integral part of *T. cruzi* telomere. Chiurillo et al. (2017) have proposed that the 189 bp junction may act as a seed to stabilize and/or create new telomeres via telomerase, but it is also possible that it was adopted as a recognition site for other proteins that contribute to the telomeric function.

## Gene Families and Subtelomeres

In *T cruzi* apart from the TS family other gene families suffered big expansions, namely RHS, DFG-1, MASP, and MUC and these genes tend to form clusters at multiple locations in the genome, in some cases covering whole chromosomes (El-Sayed et al., 2005; Weatherly et al., 2009; Berna et al., 2018; Callejas-Hernández et al., 2018). Except for MUC and MASP families, these expanded families are often found at chromosomal terminal locations (El-Sayed et al., 2005; Weatherly et al., 2009; Moraes Barros et al., 2012; Callejas-Hernández et al., 2018). Therefore it is not surprising to detect members of these families when *T. cruzi* telomeres and subtelomeres are cloned (Chiurillo et al., 1999; Freitas-Junior et al., 1999). Thus, the *T. cruzi* genome is organized in blocks of syntenic non-repeated gene sequences at more interstitial locations, and non-syntenic blocks consisting of repeated genes that are intermingled with retrotransposons and other highly repeated elements, some of which are located at subtelomeres (El-Sayed et al., 2005). The presence of many repeated genes may propitiate recombination events that, although not desirable for housekeeping genes, favor the generation of variability for genes coding for surface proteins.

The uniqueness of terminal chromosomal locations (subtelomeres) as a ground for the generation of new capabilities has been well documented among other organisms in yeast (Haber, 1998), *Plasmodium* (Scherf et al., 2008), *T. brucei* (Horn, 2004), and the expansion of human odor receptors (Mefford et al., 2001). Based on these observations and in the composition of *T. cruzi* telomeres and subtelomeres, we proposed that subtelomeres were places where the variability of some of these multigene families was generated, and in later events, the gene variants were mobilized to different locations in the genome (Kim et al., 2005).

## Hypothetical Mechanisms to Generate Gene Variability

What are the mechanisms that may generate gene variability in *T. cruzi*, and how has the mobilization of gene variants occurred? Is the variability generated by gene conversion or unequal crossing over? Are these processes still occurring in *T. cruzi* populations?

Our first attempt to address these questions was through *in silico* simulation studies using as inputs real gene sequences generated by the *T. cruzi* genome project (Azuaje et al., 2007). The simulations evaluated the generation of variability by introducing different mutagenic pressures in housekeeping genes, and members of gene families with presence at subtelomeres. The premise was that mutation rates would be a trade-off between the generation of variability to expand adaptive capabilities, and the need to keep important core functions. This study concluded that housekeeping genes were more robust against the introduction of random point mutations than genes coding for surface proteins and that the most effective mechanism to introduce variability was gene conversion. The energetic burden of keeping a large number of pseudogenes is an indication that they play an important role in the parasite, an observation that prompted us to include pseudogenes of RHS, TS, and DGF-1 in our simulations. The results confirmed the potential of pseudogenes to contribute to the generation of variability. This finding contradicts the idea that pseudogenes are merely relics of gene deterioration (Rogers et al., 2011) or pseudogenization.

An important piece of information came from experiments using *T. cruzi* artificial chromosomes (pTAC) carrying the 189 bp junction, hexameric repeats, and drug selection markers (Curto et al., 2014). When *T. cruzi* epimastigotes were transformed with these pTCAs, they were able to replicate with surprising stability for 150 generations in the presence of the selection drug, or 60 generations without it. In other words, the pTACs showed non-detectable sequence exchange with the host chromosomes and replicated and segregated without the presence of centromeres, or perhaps the telomere was fulfilling this role. In a more recent experiment (Chiurillo et al., 2016b), addressed the possibility of chromosomal sequence exchanges by introducing a cutting site for the rare meganuclease I-SceI within the RHS gene of one pTACs (pTAC-D6CISceI^*^) harboring larger portions of *T. cruzi* subtelomeres. After transforming this pTAC into *T. cruzi* cells expressing the meganuclease I-SceI, and confirming that double-strand breaks (DBSs) were produced, the probing pTAC was examined to check whether the DSBs were repaired. Out of seven clones studied, six showed repairs, as evidenced by the disappearance of the I-SecI site, and the reversion to the original pTAC (pTAC-D6C^*^). The most likely explanation is that the repair events used as template the host subtelomere homologous to the pTAC. The seventh clone was repaired losing the I-SecI site, but the sequences around the repair site shared homology with the subtelomere of another chromosome (ectopic recombination). From these experiments several conclusions can be derived: first chromosomal exchanges at the subtelomere can be promoted by the introduction DSBs; second the repair mechanism for these DSBs is homologous recombination (HR), or some version of it (Dressen et al., 2007); third, events involving exchanges with non-homologous chromosomes (ectopic recombination) can occur, with the possibility of generating gene variants. These experiments don't rule out that similar exchanges can occur at more interstitial locations. The absence of detectable recombination in earlier pTAC experiments was likely due to a lack of sufficient homology with any given chromosome subtelomere, and/or that DSBs are strictly necessary to induce recombination.

Contrary to most eukaryotes in *T. brucei* and *T. cruzi* the most important repair mechanism for DSBs repair is HR, with minor participation of microhomology repair (MHR). To detect MHR-DSB repair, it was necessary to abolish HR (Glover et al., 2008).

In *T. cruzi* out of the potential genes that participate in HR, RAD51 plays a central role in facilitating homologous strand invasion (Gomes Passos Silva et al., 2018). An interesting activity discovered after massive gamma radiation of *T. cruzi* cells was tyrosyl-DNA phosphodiesterase I (Tdr-I), an enzyme that plays an important role in Topoisomerase I mediate DNA DBS repair (Das et al., 2010). No genes for NHEJ have been found in *T. cruzi* (Gomes Passos Silva et al., 2018). MHR seems to occur in chromosomal rearrangements when single DSB is introduced by Cas9 endonuclease (Lander et al., 2015; Soares Medeiros et al., 2017).

An efficient HR repair mechanism in *T. cruzi* may explain the rapid karyotype and cell growth recovery after massive irradiation with 500 Gy of gamma radiation (Garcia et al., 2016), and the difficulty in generating mutations (indels) in CRISPR experiments involving a single DSB vs. gene replacement (Lander et al., 2015; Soares Medeiros et al., 2017). But also, the prevalence of a very stringent HR repair mechanism in genomes with a large number of repeated sequences, like *T. cruzi*, hampers (but does not eliminate) chromosomal-internal recombination events that can be detrimental to the organism.

Thus, we believe that an important source for the generation of variability in some *T. cruzi* surface proteins is subtelomeric recombination promoted by DSBs followed by a dispersion of these variants either by transpositions or ectopic recombination events. Further duplications events and genetic drift produced the current clusters that we see at several locations of the *T. cruzi*'s genome.

Along this line, experiments with meganuclease I-Sce-I in *T. brucei* (Dressen et al., 2007) revealed that the introduction of a DSB in telomeric VSG gene promoted antigenic switching via gene conversion. The break is resolved by a replication mechanism induced by this break (Break Induced Replication, BIR). Similar detailed studies to determine how DBSs repair occurs in *T. cruzi* are missing due in part to the lack of an RNAi machinery and/or inducible promoters coupled to CRISPR-Cas9 vectors.

As mentioned before, despite this stringent HR mechanism interstitial chromosome rearrangement leading to karyotype changes can occur via ectopic recombination within multigene families through duplicated sites flanking these sequences (Figures 1A,B).

## How DSBs Can Be Introduced in *T. cruzi* Subtelomeres?

In *T. cruzi* retrotransposon elements occupy nearly 5% of its genome and among these elements, the L1Tc non-LTR retrotransposon has the necessary machinery for its mobilization (Macías et al., 2018). L1Tc genome codes for an AP endonuclease activity (NL1Tc) capable of introducing breaks for the insertion of the retrotransposon, but also plays a role in repairing DSBs produced by daunorubicin (Olivares et al., 2013). Other retrotransposon elements like SLAKS and CZAR code for site-specific endonucleases (Macías et al., 2018). So, some DSBs may be introduced in the subtelomeres by retrotransposon nucleases, and HR repairs the break using as templates homologous chromatids, or on occasion non-homologous chromatids (ectopic recombination). In *T. cruzi* there are active L1Tc transposons and no RNAi machinery to counteract their activities. Several observations about the organization of multigene families reveal close associations with retrotransposon sequences i.e., most DGF-1 and TSII copies are flanked by RHS (Olivares et al., 2000; Kim et al., 2005) or L1Tc retrotransposons sequences, the duplications of RHS and L1Tc genes at both sides of these genes suggest the occurrence of ectopic recombination events (Figure 1; Olivares et al., 2000). Although L1Tc appears to be randomly distributed in *T. cruzi'*s genome, 50% of its copies are associated with RHS genes flanked by the putative insertion site 5′-TGCAGACAT-OH-3′ (Olivares et al., 2000; Figures 1B,C). Also, they are found inserted downstream the sequence GA (x)_2_AxGa (x)_5_txTATG↑A(x) _11_↑ where arrows mark the single strand cleavage sites (El-Sayed et al., 2005; Bringaud et al., 2006). How frequent DBSs leading to genetic recombination and gene variability occurs is difficult to assess, since as shown in the experiments with meganuclease I-Sce-I (Chiurillo et al., 2016b), DBSs are mainly repaired by HR using as template homolog chromosomes.

The MASP superfamily is associated with the site-specific retrotransposon TcTREZO (Souza et al., 2007). These elements are frequently found flaking MASP genes, thus providing potential sites for HR. Since TcTREZO is species-specific, it must have appeared after the separation of *T. cruzi* and *T. brucei*, and it may have played an important role in the expansion of the MASP family. MASPs proteins present highly conserved N and C terminal sequences, and a variable middle region, also besides, their gene's 5′ and 3′ UTRs are highly conserved (Bartholomeu et al., 2009). So sequence evolution in this gene family is quite different from the rest of the repeated families. Interestingly other retrotransposons and members of the gp85, DFG-1, and RHS gene families frequently interrupt MASP clusters.

Duplication and mobilization of genes can also occur via piggybacking the retrotransposon reverse transcriptase machinery by the transduction of genes neighboring the retrotransposons insertion site (Figure 1D). The termination signal for transposon transcriptases is usually weak, thus transcription can run through neighboring segments which can be duplicated and mobilized elsewhere (Xing et al., 2006). So far no experiments have been conducted to address this type of event in *T. cruzi*, although the size of the DNA segments that non-LTR retrotransposons can transduct is usually small (1 or 3 Kbp), making it an, unlike mechanism to mobilize large genes like DGF-1 (>10 Kbp).

In *T. brucei*, in the case of non-recombinational gene conversion for antigenic switching, alternatives for the generation of subtelomeres DSBs have been proposed, such as accelerated transcription, conflicts between replication and transcription machineries (da Silva et al., 2018), and TERRA (Telomeric Repeat-containing RNA) transcription leading to the formation of R-loops (Nanavaty et al., 2017; Saha et al., 2019). Since antigenic variation is a vital phenomenon for the survival of *T. brucei* populations, it is not surprising that redundant mechanisms exist to make sure that antigenic switching occurs. In the case of *T. cruzi*, not such extensive studies have been done, and it is possible that R-loops formation or transcription-replication conflicts may also contribute to the generation of subtelomere DBSs. In this review, I favored the role of retrotransposons in the generation of DBSs, given the ubiquity of these elements in the *T. cruzi* genome, their close association with important surface antigens families, and the absence of RNAi machinery.

## Conclusions

Once *T. cruzi* telomeres were fixed, members of the TS II family positioned at the subtelomeres co-evolved to be part of the transition to the telomeric repeat. Although the reason for the fixation of this junction is still unknown, it suggests a potential telomeric function for this region. The variability of some surface proteins and their localization at the subtelomeres together with retrotransposon elements suggests that these regions are grounds for the generation genetic variability. We propose that DBSs introduced in the subtelomeres by retrotransposon nucleases are repaired by homologous recombination, and when the repair includes non-homologous chromatids there is a possibility to generate gene variants. These variants are mobilized elsewhere either by transposition or ectopic recombination. Gene families increased their numbers by gene duplication to achieve higher expression levels. MUC and MASP superfamilies likely evolved in later events not related to the subtelomeres.

## Author Contributions

The author confirms being the sole contributor of this work and has approved it for publication.

### Conflict of Interest

The author declares that the research was conducted in the absence of any commercial or financial relationships that could be construed as a potential conflict of interest.

## References

[B1] Azuaje F. J. Ramirez J. L. Franco Da Silveira J. (2007). *In silico*, biologically-inspired modeling of genomic variation generation in surface proteins of *Trypanosoma cruzi*. Kinetoplastid Biol. Dis. 6, 1–12. 10.1186/1475-9292-6-6 17623100 PMC1965468

[B2] Baird D. M. (2018). Telomeres and genomic evolution Phil. Trans. R. Soc. B 373:20160437 10.1098/rstb.2016.0437 PMC5784058 29335376

[B3] Bartholomeu D. C. Cerqueira G. C. Leao A. C. A. daRocha W. D. Pais F. S. Macedo C.. (2009). Genomic organization and expression profile of the mucin-associated surface protein (masp) family of the human pathogen *Trypanosoma cruzi*. Nucleic Acids Res. 37, 3407–3417. 10.1093/nar/gkp172 19336417 PMC2691823

[B4] Berna L. Rodriguez M. Chiribao M. L. Parodi-Talice A. Pita S. Rijo G.. (2018). Expanding an expanded genome: long-read sequencing of *Trypanosoma cruzi*. Microb. Genom. 4:e00017. 10.1099/mgen.0.000177 29708484 PMC5994713

[B5] Bringaud F. Bartholomeu D. C. Blandin G. Delcher A. Baltz T. El-Sayed N. M.. (2006). The *Trypanosoma cruzi* L1Tc and NARTc non-LTR retrotransposons show relative site-specificity for insertion. Mol. Biol. Evol. 23, 411–420. 10.1093/molbev/msj046 16267142

[B6] Bringaud F. Biteau N. Melville S. E. Hez S. El-Sayed N. M. (2002). A new, expressed multigene family containing a hot spot for insertion of retroelements are associated with polymorphic subtelomeric regions of *Trypanosoma brucei* Euk. Cell 1, 137–151. 10.1128/EC.1.1.137-151.2002 PMC118050 12455980

[B7] Briones M. R. Egima C. M. Eichinger D. Schenkman S. (1995). Trans-sialidase genes expressed in mammalian forms of Trypanosoma cruzi evolved from ancestor genes expressed in insect forms of the parasite. J. Mol. Evol. 41, 120–131. 10.1007/BF00170663 7666441

[B8] Callejas-Hernández F. Rastrojo A. Poveda C. Gironès N. Fresno M. (2018). Genomic assemblies of newly sequenced *Trypanosoma cruzi* strains reveal new genomic expansion and greater complexity. Sci. Rep. 8:14631. 10.1038/s41598-018-32877-2 30279473 PMC6168536

[B9] Cavalier-Smith T. (2002). The phagotrophic origin of eukaryotes and phylogenetic classification of Protozoa. Int. J. Syst. Evol. Microbiol. 52, 297–354. 10.1099/00207713-52-2-297 11931142

[B10] Chiurillo M. A. Antonio C. R. Mendes Marini M. Torres de Souza R. Franco Da Silveira J. (2017). Chromosomes ends and the telomere biology in Trypanosomatids. Front. Parasitol. 1, 104–133. 10.2174/9781681084053117010006

[B11] Chiurillo M. A. Cano I. Franco Da Silveira J. Ramirez J. L. (1999). Organization of telomeric and sub-telomeric regions of chromosomes from the protozoan parasite *Trypanosoma cruzi*. Mol. Biochem. Parasitol. 100, 173–183. 10.1016/S0166-6851(99)00047-X 10391379

[B12] Chiurillo M. A. Cortez D. R. Lima F. M. Cortez C. Ramírez J. L. Martins A. G.. (2016a). The diversity and expansion of the trans-sialidase gene family is a common feature in *Trypanosoma cruzi* clade members. Infect. Genet. Evol. 37, 266–274. 10.1016/j.meegid.2015.11.024 26640033

[B13] Chiurillo M. A. Moraes Barros R. R. Souza R. T. Marini M. M. Antonio C. R. Cortez D. R.. (2016b). Subtelomeric I-SceI-mediated double-strand breaks are repaired by homologous recombination in *Trypanosoma cruzi*. Front. Microbiol. 7:2041. 10.3389/fmicb.2016.02041 28066363 PMC5177640

[B14] Chiurillo M. A. Santos M. R. M. Franco Da Silveira J. Ramirez J. L. (2002). An improved general approach for cloning and characterizing telomeres: the protozoan parasite *Trypanosoma cruzi* as a model organism. Gene 294, 197–204. 10.1016/S0378-1119(02)00768-0 12234681

[B15] Curto M. L. de Lorenzi H. A. Moraes Barros R. R. Souza R. Levin M. J. Da Silveira J. F. (2014). Cloning and expression of transgenes using linear vectors in *Trypanosoma cruzi*. Int. J. Parasitol. 44, 447–456. 10.1016/j.ijpara.2014.03.009 24759431

[B16] da Silva M. S. Hovel-Miner G. A. Briggs E. M. Elias M. C. McCulloch R. (2018). Evaluation of mechanisms that may generate DNA lesions triggering antigenic variation in African trypanosomes. PLoS Pathog. 14:e1007321. 10.1371/journal.ppat.1007321 30440029 PMC6237402

[B17] Das B. B. Dexheimer T. S. Maddali K. Pommier Y. (2010). Role of tyrosyl-DNA phosphodiesterase (TDP1) in mitochondria. PNAS 107, 19790–19795. 10.1073/pnas.1009814107 21041670 PMC2993338

[B18] Di Noia J. M. Sanchez D. O. Frasch A. C. (1995). The protozoan *Trypanosoma cruzi* has a family of genes resembling the mucin genes of mammalian cells. J. Biol. Chem. 270, 24146–24149. 10.1074/jbc.270.41.24146 7592617

[B19] Dressen O. Li B. Cross G. A. M. (2007). Telomere structure and function in trypanosomes: a proposal. *Nat. Rev*. Microbiol. 5, 70–75. 10.1038/nrmicro1577 17160000

[B20] El-Sayed N. M. Myler P. J. Bartholomeu D. C. Nilsson D. Aggarwal G. Tran A.-N. (2005). The genome sequence of *Trypanosoma cruzi*, the etiological agent of Chagas' disease. Science 309, 410–415. 10.1126/science.1112631 16020725

[B21] Freitas L. M. Lopes dos Santos S. Rodrigues-Luiz G. F. Mendes T. A. O. Rodrigues T. S. Gazzinelli R. T.. (2011). Genomic analyses, gene expression and antigenic profile of the trans-sialidase superfamily of *Trypanosoma cruzi* reveal an undetected level of complexity. PLoS ONE 6:e25914. 10.1371/journal.pone.0025914 22039427 PMC3198458

[B22] Freitas-Junior L. Marques Porto R. Pirrit L. A. Schenkman S. Scherf A. (1999). The identification of the telomeres in *Trypanosoma cruzi* reveals highly heterogeneous lengths in different parasite strains. Nucleic Acids Res. 12, 2451–2456. 10.1093/nar/27.12.2451 PMC148447 10352173

[B23] Fulneckova J. Ševčíková T. Falkus J. Lukešová A. Lukeš M. Vlček C.. (2013). A broad phylogenetic survey unveils the diversity and evolution of telomeres in eukaryotes. Gen. Biol. Evol. 5, 468–483. 10.1093/gbe/evt019 23395982 PMC3622300

[B24] Garavis M. Gonzalez C. Villasante A. (2013). On the origin of the eukaryotic chromosome: the role of noncanonical DNA structures in telomere evolution. Genome Biol. Evol. 5, 1142–1150. 10.1093/gbe/evt079 23699225 PMC3698924

[B25] Garcia J. B. F. Vieira da Rocha P. Costa-Silva H. M. Alves C.eres C. L. Machado C. R. Cruz A.K. (2016). *Leishmania major* and *Trypanosoma cruzi* present distinct DNA damage responses. Mol. Biochem. Parasitol. 207, 23–32. 10.1016/j.molbiopara.2016.05.004 27188657

[B26] Glover L. McCulloch R. Horn D. (2008). Sequence homology and microhomology dominate chromosomal double-strand break repair in African trypanosomes. Nucleic Acids Res. 36, 2608–2618. 10.1093/nar/gkn104 18334531 PMC2377438

[B27] Gomes Passos Silva D. da Silva Santos S Nardelli S. C. Mendes I. C. Guimarães Freire N. C. Marcal Repolês B.. (2018). The *in vivo* and *in vitro* roles of *Trypanosoma cruzi* Rad51 in the repair of DNA double-strand breaks and oxidative lesions. PLOS Neg. Trop. Dis. 12:e0006875. 10.1371/journal.pntd.0006875 30422982 PMC6258567

[B28] Gonzalez A. M. Azuaje F. J. Ramirez J. L. Da Silveira J. F. Dorronsoro J. R. (2009). Machine learning techniques for automated classification of adhesine-like proteins in *Trypanosoma cruzi*. IEEE/ACM Trans. Comp. Biol. Bioinf. 6, 695–702. 10.1109/TCBB.2008.125 19875867

[B29] Haber J. E. (1998). Mating-Type Gene Switching in *Saccharomyces cerevisiae*. Annu. Rev. Genet. 32, 561–599. 10.1146/annurev.genet.32.1.561 9928492

[B30] Horn D. (2004). The molecular control of antigenic variation in *Trypanosoma brucei*. Curr Mol. Med. 4, 563–574. 10.2174/1566524043360078 15357208

[B31] Kawashita S. Y. Da Silva C. V. Mortara R. A. Burleigh B. Briones M. R. S. (2009). Homology, paralogy, and function of dgf-1, a highly disperse *Trypanosoma cruzi* specific gene family and its implications for information entropy of its encoded proteins. Mol. Biochem. Parasitol. 165, 19–31. 10.1016/j.molbiopara.2008.12.010 19393159

[B32] Kim D. Chiurillo M. A. El-Sayed N. M. Jones K. Santos M. M. R. Porcile P. E.. (2005). Telomere and subtelomere of *Trypanosoma cruzi* chromosomes are enriched in (pseudo) genes of retrotransposon hot spot and trans-sialidase-like gene families: the origins of *T. cruzi telomeres*. Gene 346, 153–161. 10.1016/j.gene.2004.10.014 15716016

[B33] Lander N. Bernal C. Diez N. Añez N. Docampo R Ramirez J. L. (2010). Localization and developmental regulation of a disperse gene family 1 protein in *Trypanosoma cruzi*. Infect. Immun. 78, 231–241. 10.1128/IAI.00780-09 19841080 PMC2798230

[B34] Lander N. Li Z.-H. Niyogi S. Docampo R. (2015). CRISPR/Cas9-induced disruption of paraflagellar rod protein 1 and 2 genes in *Trypanosoma cruzi* reveals their role in flagellar attachment. MBio 6:e01015. 10.1128/mBio.01012-15 26199333 PMC4513075

[B35] Macías F. Afonso-Lehmann R. López M. C. Gómez I Thomas M. C. (2018). Biology of *Trypanosoma cruzi* retrotransposons: from an enzymatic to a structural point of view. Curr. Genom. 19, 110–118. 10.2174/1389202918666170815150738 29491739 PMC5814959

[B36] Mefford H. C. Linardopoulou E. Coil D. van den Engh G. Trask B. J. (2001). Comparative sequencing of a multicopy subtelomeric region containing olfactory receptor genes reveals multiple interactions between non-homologous chromosomes. Hum. Mol. Genet. 10, 2363–2372. 10.1093/hmg/10.21.2363 11689483

[B37] Millar A. E. Wleklinski-Lee M. Kahn S. J. (1999). The surface Protein superfamily of *Trypanosoma cruzi* stimulates a solarized Th1 response that Becomes anergic. *J*. Immunol. 162, 6092–6099. 10229851

[B38] Moraes Barros R. R. Marini M. M. Antônio C. R. Cortez D. R. Miyake A. M. Lima F. M.. (2012). Anatomy and evolution of telomeric and subtelomeric regions in the human protozoan parasite *Trypanosoma cruzi*. BMC Genom. 13:229. 10.1186/1471-2164-13-229 22681854 PMC3418195

[B39] Nanavaty V. Sandhu R. Jehi S. G. Pandya U. M. Li B. (2017). *Trypanosoma brucei* RAP1 maintains telomere and subtelomere integrity by suppressing TERRA and telomeric RNA: DNA hybrids. Nucleic Acids Res. 45, 5785–5796. 10.1093/nar/gkx184 28334836 PMC5449629

[B40] Olivares M. Lopez M. C. Garcia-Perez J. L. Briones P. Pulgar M. Thomas M. C. (2013). The endonuclease NL1Tc encoded by the LINE L1Tc from *Trypanosoma cruzi* protects parasites from daunorubicin DNA damage. Biochim. Biophys. Acta 1626, 25–32. 10.1016/S0167-4781(03)00022-8 12697326

[B41] Olivares M. Thomas M. C. Lopez-Barajas A. Requena J. M. Garcia-Perez J. L. Angel S. (2000). Genome clustering of the *Trypanosoma cruzi* nonlong terminal L1Tc retrotransposon with defined intersperse repeated DNA elements. Electrophoresis 21, 2973–2982. 10.1002/1522-2683(20000801)21:14&lt;2973::AID-ELPS2973&gt;3.0.CO;2-4 11001312

[B42] Peterson D. S. Fouts D. L. Manning J. E. (1989). The 85-kd surface antigen gene of the *Trypanosoma cruzi* is telomeric and a member of a multigene family. EMBO J. 8, 3911–3916. 10.1002/j.1460-2075.1989.tb08571.x 2684649 PMC402082

[B43] Podlevsky J. D. Chen J.-L. (2016). Evolutionary perspectives of telomerase RNA structure and function. RNA Biol. 13, 720–732. 10.1080/15476286.2016.1205768 27359343 PMC4993307

[B44] Rogers M. B. Hilley J. D. Dickens N. J. Wilkes J. Bates P. A. Depledge D. P.. (2011). Chromosome and gene copy number variation allow structural change between species and strains of *Leishmania major*. Gen. Res. 21, 2129–2142. 10.1101/gr.122945.111 22038252 PMC3227102

[B45] Roggentin P. Schauer R. Hoyer L. L. Vimr E. R. (1993). The sialidase superfamily and its spread by horizontal gene transfer. *Mol*. Microbiol. 9, 915–921. 10.1111/j.1365-2958.1993.tb01221.x 7934919

[B46] Ruef B. J. Dawson B. D. Tewari D. Fouts D. L. Manning J. E. (1994). Expression and evolution of members of the *Trypanosoma cruzi* trypomastigote surface antigen multigene family. Mol. Biochem. Parasitol. 63, 109–120. 10.1016/0166-6851(94)90013-2 8183309

[B47] Saha A. Nanavaty V. P. Li B. (2019). Telomere and subtelomere R-loops and antigenic variation in trypanosomes. J. Mol. Biol. 10.1016/j.jmb.2019.10.025. [Epub ahead of print]. 31682833 PMC7195242

[B48] Scherf A. Lopez-Rubio J. J. Riviere L. (2008). Antigenic variation in *Plasmodium falciparum*. Annu. Rev. Microbiol. 62, 445–470. 10.1146/annurev.micro.61.080706.093134 18785843

[B49] Schwerdtfeger S. M. Melzig M. F. (2010). Sialidases in biological systems Pharmazie 65, 551–561. 10.1002/chin.201047267 20824954

[B50] Soares Medeiros L. C. South L. Peng D. Bustamante J. M. Wang W. Bunkofske. (2017). Rapid, selection-free, high-efficiency genome editing in protozoan parasites using CRISPR-Cas9 ribonucleoproteins. MBio 8, e01788–17. 10.1128/mBio.01788-17 29114029 PMC5676044

[B51] Souza R. T. Santos M. R. M. Lima F. M. El-Sayed N. M. Myler P. J. Ruiz J. C.. (2007). New *Trypanosoma cruzi* repeated element that shows site specificity for insertion eukaryt. Cell 6, 1228–1238. 10.1128/EC.00036-07 17526721 PMC1951114

[B52] Stevens J. R. Noyes H. A. Dover G. A. Gibson W. C. (1999). The ancient and divergent origins of the human pathogenic trypanosomes, *Trypanosoma brucei* and *T. cruzi*. Parasitology 118, 107–116. 10.1017/S0031182098003473 10070668

[B53] Weatherly D. B. Boehlke C. Tarleton R. L. (2009). Chromosome level assembly of the hybrid *Trypanosoma cruzi* genome. BMC Genomics 10:255. 10.1186/1471-2164-10-255 19486522 PMC2698008

[B54] Xing J. Wan G. Belancio V. P. Cordaux R. Deininger P. L. Batzer M. A. (2006). Emergence of primate genes by retrotransposon mediated sequence transduction PNAS 103, 17608–17613. 10.1073/pnas.0603224103 17101974 PMC1693794

